# Phenotypic and genotypic characteristics of tetracycline resistant *Acinetobacter baumannii* isolates from nosocomial infections at Tehran hospitals

**Published:** 2014-01

**Authors:** Mohammad Hossein Maleki, Zamberi Sekawi, Setareh Soroush, Farid Azizi-Jalilian, Khairollah Asadollahi, Sattar Mohammadi, Mohammad Emaneini, Morovat Taherikalani

**Affiliations:** 1 Clinical Microbiology Research Center, Ilam University of Medical Sciences, Ilam, Iran; 2 Department of Medical Microbiology and Parasitology, Faculty of Medicine and Health Sciences, University of Putra, Malaysia; 3 Department of Microbiology, School of Medicine, Ilam University of Medical Sciences, Ilam, Iran; 4 Psychosocial Injuries Research Center, Ilam University of Medical Sciences, Ilam, Iran; 5 Department of Microbiology, School of Medicine, Tehran University of Medical Sciences, Tehran, Iran

**Keywords:** *Acinetobacter baumannii*, REP-PCR, *Tet A*, *Tet B*, Tetracycline resistance

## Abstract

***Objective(s):*** To date, the most important genes responsible for tetracycline resistance among *Acinetobacter baumannii* isolates have been identified as *tet *A and *tet *B. This study was carried out to determine the rate of resistance to tetracycline and related antibiotics, and mechanisms of resistance.

***Materials and Methods:*** During the years 2010 and 2011, a total of 100 *A. baumannii* isolates were recovered from patients in different hospitals of Tehran, Iran. Antimicrobial susceptibility to tetracycline, minocycline, doxicycline and tigecycline was evaluated by E-test. Polymerase chain reaction (PCR) of the* tet *A and *tet *B genes was performed using specific primers, after which the isolates were subjected to Repetitive Extragenic Palindromic-PCR (PCR) to identify the major genotypes.

***Results:*** Of all isolates, 89% were resistant to tetracycline (MIC_50 _= 32 µg/ml, MIC_90_ = 512 µg/ml). Minocycline with the resistant rate of 35% (MIC_50_ = 16 µg/ml, MIC_90 _=32 µg/ml) and doxicycline with the resistant rate of 25% (MIC_50_ = 16 µg/ml, MIC_90_= 32 µg/ml) have a good activity against* A. baumannii* isolates. All isolates were sensitive to tigecycline. Frequencies of *tet *B and *tet *A genes and coexistence of *tet *A and *tet *B among the isolates resistant to tetracycline, were 87.6%, 2.2% and 1.1%, respectively. Distribution of REP-types among *A. baumannii *isolates was types A (40%), B (30%), C (10%), D (5%) and E (5%)**.**

***Conclusion:*** It seems that *tet *A and *tet *B genes play an important role in the induction of resistance towards tetracyclines used in this study. It is suggested that further studies focus on other antimicrobial drugs and combinations in order to achieve a successful therapy against multi drug resistance (MDR) *A. baumannii* strains in Iran.

## Introduction

*Acinetobacter baumannii* is the most clinically important species in the Acinetobacter genus that is involved in 1-2% of nosocomial pneumonia primarily in debilitated patients (1, 2). Infections due to *A. baumannii*, especially multi drug resistant strains, have been reported worldwide, so that only a few antibiotics have remained effective against MDR strains. 

Resistance against aminopenicillins, ureidop-enicillins, cephalosporins, cephamycins, aminoglyc-osides, chloramphenicol, tetracyclines, fluoroquinol-ones and carbapenems were initially reported from different parts of the world (2). Tetracycline is a bacteriostatic antibiotic which inhibits protein synthesis by preventing the attachment of aminoacyl-tRNA to the ribosomal acceptor site. To date, the main mechanisms responsible for tetracycline resistance have been identified as (i) expression of efflux pumps and (ii) ribosomal protection. *tet *A and *tet *B are the most extensively characterized genes responsible for ribosomal protection. Although the distribution of many genes responsible for resistance towards antimicrobial agents especially carbapenems and aminoglycosides, has been previously reported among *A. baumannii* isolates in Iran (3-12).

There appears to be no comprehensive data concerning the mechanisms of resistance towards tetracycline and related antibiotics among *A. baumannii* isolates in this country. The aim of the present study was, therefore, to analyze the molecular mechanisms of resistance towards tetracycline and related antibiotics among clinical isolates of *A. baumannii* and to estimate the prevalence of the genetic constructs containing *tet *A and *tet *B genes in the isolates of this microorganism. Furthermore, the susceptibility of the isolates towards tigecycline (as a glycylcycline and a derivative of minocycline) and tetracyclines including tetracycline, minocycline and doxicycline was surveyed in this study.

## Materials and Methods

***Study population***

A total of 100 non-repetitive *A. baumannii* isolates were recovered in years 2010 and 2011 from patients in different hospitals of Tehran, Iran. The isolates were non-repetitive, meaning that each isolate was obtained from a particular patient and each patient was sampled only once. These isolates were cultured from wounds (n = 40), the trachea (n = 30), blood (n = 10), urine (n = 15), andcatheter (n = 5). Strains were isolated mainly from patients in intensive care units (n = 36), burned ward (n = 30), internal ward (n = 20) and surgery (n = 4) from eight hospitals in Tehran, Iran.

 All the isolates were identified as *A. baumannii* by the detection of *bla*_OXA-51-like_, an intrinsic and species specific gene, using API 20NE and biochemical testing according to those reported in previous reports (13).

***Antimicrobial susceptibility***

Using E-test, the susceptibility of all the isolates toward tetracycline, minocycline, doxicycline and tigecycline was tested. The isolates were then interpreted according to the manufacturer instructions and CLSI guidelines (14). *Escherichia coli*, ATCC 25922 and *Pseudomonas aeruginosa, *ATCC 27852 were used as internal control. MIC interpretative standards for together tetracycline, minocycline and doxicycline was S: ≤ 4μg/ml, I: 8 μg/ml and R: ≥16 μg/ml, respectively. The criteria defined by the US Food and Drug Administration (FDA) for Enterobacteriaceae were used for tigecycline (≤2, ≥4 μg/ml for susceptible and non susceptible strains, respectively) (15). 

***DNA extraction***

Strains were maintained at -70°C in 80% /20% (v/v) glycerol in LB medium to preserve genetic variation during storage and were grown overnight on MacConkey agar at 37°C. DNA extraction was carried out by DNA Extraction kit (BIONER, Republic of Korea) in accordance with the manufacturer instructions. DNA was stored at -20°C until further use.

***PCR amplification of tet A and tet B genes***

The PCR products for *tet A* and *tet *B genes were considered presumptive positive based on amplicon sizes of 164bp and 206 bp, respectively. In the case of a negative PCR, PCR amplifications were repeated at least twice for these genes. A negative control was run along each PCR.

The primer sequences included *tet A* (F: 5'-GCG CGATCTGGTTCACTCG-3'; R: 5'-AGTCGACAGYRGCG CCGGC-3') and *tet B* (F: 5'- CGTGAATTTATTGCTT CGG-3'; R: 5'-ATACAGCATCCAAAGCGCAC-3') (16). PCR were performed in a final volume of 25 μl containing 1X PCR buffer, 2 mM MgCl_2_, 2 mM dNTPs, 10 pmol of primers, 0.25 U Taq DNA polymerase (Fermentas, UK) and 5 μl of template DNA.

PCR conditions included 30 cycles of ampli-fication under the following conditions: denaturation at 95^°^C for 30 s, annealing at 56^°^C for 1 min, and extension at 72^°^C for 1 min/kb product. Cycling was followed by a final extension at 72^°^C for 10 min. PCR products were resolved on 1.0% agarose gels, stained with ethidium bromide, and photographed with UV illumination. The 100-bp DNA ladder was used to assess PCR product size. 

***REP-PCR***

All the isolates were subjected to REP-PCR typing method for finding common REP-types among all of the isolates. The primers used for REP-typing include F: 5'- IIIGCGCCGICATCAGGC-3' and R: 5'-ACGTCTTATCAGGCCTAC- 3' (17). Amplification reaction was performed in a final volume of 25μl. The reaction contain 2.5 μl of 10X PCR buffer, 1.25U Taq DNA polymerase (Fermentas, UK), 0.8 μl of 2 mM mixed dNTP,1.5 μl of 25 mM MgCl_2_, 1 μl of 10 pmol of primers and 5 μl of template DNA. Amplification reaction was carried out by thermal cycler (Eppendorf, Germany) with an initial denaturation at 94^°^C for 10 min, followed by 30 cycles of denaturation at 94^°^C for 1 min, annealing at 45^°^C for 1 min, and extension at 72^°^C for 1 min, followed by final extension at 72^°^C for 16 min. Samples (20 µl) of each PCR end-product were analysed on agarose 2% wlv gels.

## Results

Tigecycline, being effective against all tested *A. baumannii* isolates (100/100), was the most effective antimicrobial agent with the MIC_50_ = 0.125 µg/ml to MIC_90_ = 2 µg/ml. Resistance rate against doxicycline, minocycline and tetracycline was 18%, 19% and 80%, respectively (Table 1, 2). Totally, 50 % (n =50/100) and 4% (n = 4/100) of all the strains were only resistant to tetracycline and minocycline, respectively. Resistance rates of combinations of tetracycline + minocycline, tetracycline + minocy-cline + doxicycline, tetracycline + doxicycline, and minocycline + doxicycline were 16% (n = 16/100), 13% (n = 13/100), 10% (n = 10/100) and 2% (n = 2/100), respectively. Among the isolates, 4 (n = 4/100; 4%) were sensitive to all the antibiotics used in this study whilst 96 (n = 96/100; 96%) were resis-tant to at least one antibiotic among which 13 (n = 13/96; 13.54%) were resistant to all the antibiotics used in this study. Among the resistant isolates, 36 (n = 36/96; 37.5%) were recovered from ICU, 30 from burns ward (n = 30/96; 31.2%), 20 from internal ward (n = 20/96; 20.8%) and 4(n = 4/96, 4.1%) from surgery ward, respectively. Nearly all tetracycline resistant isolates harbored at least one resistance gene (Table 3). *Tet *B was the most frequent encoding gene among tetracycline (n = 78/89; 87.6%), minocycline (n = 11/35; 31.4%) and doxicycline (n = 10/25; 40%) resistant *A. baumannii* isolates (Table 3). Coexistences of *tet *A + *tet *B was seen among 1.1% (1 out of 80) of the tetracycline resistant isolates, 31.2% (5 out of 16) of the minocycline resistant and 44.4% (8 out of 18) of the doxicycline resistant *A. baumannii* isolates. According to Table 3 less than 50% of intermediate isolates (intermediate resistance to tetracycline: 44.4%, intermediate resis-tance to minocycline: 31.2% and intermediate re-sistance to doxycycline: 28.5%) harbored *tet* B gene (Table 3). REP-PCR showed five clusters A, B, C, D and E with distribution rates of 40% (n = 40/100), 30% (n = 30/100), 10% (n = 10/100), 5% (n = 5/100) and 5% (5/100) among tetracycline resistant *A. baumannii* isolates, respectively. The REP patterns between ten *A. baumannii* isolates could not be dis-tinguished by REP-fingerprinting (Dendrograme 1). 

**Table 1:** Antimicrobial susceptibility patterns of tetracycline and related antibiotic against* Acinetobacter baumannii* isolates

	MIC vlues			N (%)		
Antibiotics	Ranges of MIC	MIC_50_ (µg/ml)	MIC_90_ (µg/ml)	Sensitive	Intermediate	Resistant
Tigecycline	0.125-2	0.5	2	100(100)	0	0
Tetracycline	0.125-512	32	512	11 (11%)	9 (9%)	80 (80%)
Minocycline	0.125-512	16	32	65 (65)	16 (16)	19(19)
Doxicycline	0.125-512	16	32	75 (75)	7 (7)	18 (18)

**Table 2:** MIC value of antibiotics against *Acinetobacter baumannii* isolates

Antibiotics	MIC diluted (µg/ml)												
	0.125	0.25	0.5	1	2	4	8	16	32	64	128	256	512
Tetracycline	1	0	0	2	4	4	9	19	19	5	2	5	26
Minocycline	10	5	5	10	16	19	16	3	5	2	2	3	4
Doxicycline	8	14	12	17	9	8	7	2	5	2	2	2	5
Tigecycline	21	31	28	10	10	0	0	0	0	0	0	0	0

**Table 3:** Distribution of tetracycline resistance genes among *Acinetobacter baumannii* isolates regarding different antibiotics

Antibiotics	Tetracycline n (%)		Minocycline n (%)		Doxicycline n (%)	
	I (n= 9)	R (n=80)	I (n=16)	R (n=19)	I (n=7)	R (n=18)
Gene/s						
*tet *A	0 (0)	2 (2.5)	1 (6.25)	3 (15.7)	2 (28.5)	3 (16.6)
*tet *B	4(44.4)	74 (83.1)	5 (31.2)	6 (31.5)	2 (28.5)	8 (44.4)
*tet *A + *tet *B	0 (0)	1 (1.2)	5 (31.2)	5 (26.3)	2 (28.5)	4 (22.2)
None	5 (55.5)	3 (3.7)	5 (31.2)	5 (26.3)	1 (14.2)	3 (16.6)
						

## Discussion

The current study can be considered as the first comprehensive study evaluating tetracycline resis-tant *A. baumannii* isolates containing *tet *A and *tet *B resistance determinants in different hospital of Tehran, Iran. 

In most clinical practices, tetracyclines are not commonly used as treatments against *A. baumannii* infections due to the lack of data demonstrating their therapeutic efficacy. As a result, tetracycline resistance is not routinely monitored for multi-drug resistant* A. baumannii* and very few data are avail-able on the phenotypic and genotypic characteri-zation of resistance against these bacteriostatic agents among *A. baumannii* strains in Iran. Limited data was previously reported regarding tetracycline resistance among *A. baumannii* strains isolated from burn patients in one educational hospital in Tehran, Iran (6, 8, 12). The current study was, however, carried out in different hospitals and different samples. 

Most of the *A. baumannii* strains in this study (n = 89/100; 89%) were tentatively classified as tetra-cycline resistant based on an E-test (MIC_50 _= 32_µg/ml_ and MIC_90 _= 512_µg/ml_). Resistance of the isolates was subsequently confirmed by MIC values of ≥16 μg/ml, which is the proposed clinical breakpoint of CLSI to define resistance against tetracycline, minocycline and doxicycline. 

**Dendrogram 1:**
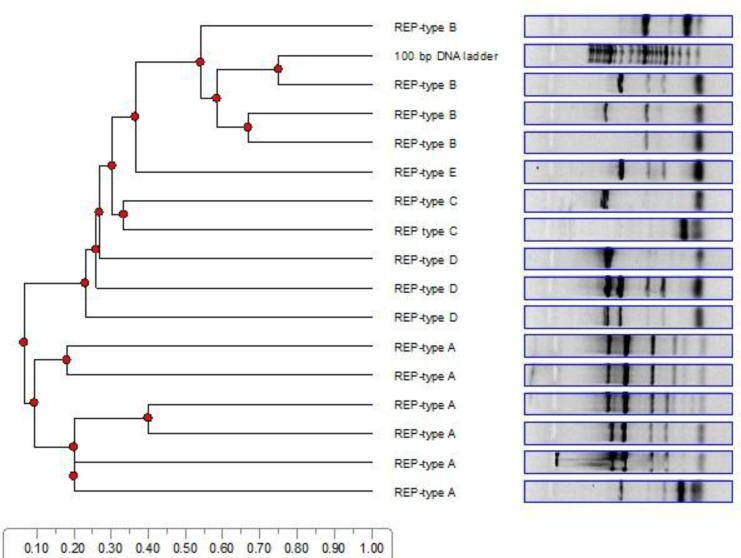
Determination of different REP-types A, B, C, D and E among *Acinetobacter baumannii* isolates and their clonal relationships by Total Lab TL120 software. Advanced analysis of 1D gel electrophoresis gel images

In this study, like the report from Iran (18), all of the isolates were sensitive to tigecycline, however, in recent reports from Iran, tetracycline resistant *A. baumannii* isolates was reported from Tehran and Tabriz (15, 19).

Tigecycline is a novel expanded broad-spectrum glycylcycline antibiotic that has a good activity against isolates which are either resistant and/or sensitive to tetracycline, minocycline and doxicyc-line. It seems that the activity of tigecycline is not affected by the tetracycline resistance mechanisms described above. Although resistance to tigecycline is not seen among *A. baumannii* isolates, it has been recently shown that resistance against these antibiotics may exist due to the over expression of AdeABC efflux pump (20-22). Both tigecycline highly resistant and sensitive *A. baumannii* have been reported from all over the world (22, 23). 

Being highly resistant to tetracycline is common among *A. baumannii *isolates and the result of this study confirmed those data (24).

 Activity of doxycycline in our Iranian *A. baumannii* isolates is in line with the reports from European countries. Mezzatesta and collageous also found that 94% of the isolates were sensitive to doxycycline (22). 

Likewise, Pei *et al* reported high degree of sensitivity to minocycline. We also found mino-cycline has a moderate activity against *A. baumannii* isolates with the sensitivity rate of 65% (25). The result of Denis *et al* study which showed MDR *A. baumannii *isolates in USA, were susceptible to tigecycline and minocycline is in consistency with our results (26).

The results, however, suggested a higher prevalence of the *tet *B gene among these clinical isolates (n = 78/89; 87.6%).

 These results are consistent with those of Guar-dabassi *et al*, who found that *tet *A and *tet *B are the genes responsible for tetracycline resistance (27).

The results of this study, therefore, supports these data, since the *tet *A gene was identified in the strains resistant to tetracycline but not in those resistant to minocycline, whilst the gene was not found in the strains resistant to both antibiotics (28). These strains may possess the *tet *B determinant, which would confer with the resistance to both antibiotics. The genes *tet *A and* tet* B were not detected in 22 clinical isolates of tetracycline resistant *A. baumannii*. Tetracycline resistance in these isolates is probably due to other resistance genes and efflux pumps (29). REP-patterns could not be distinguished in ten of the isolates studied. Furthermore, only five clusters were identified among the *A. baumannii* isolates. Patterns of resis-tance and distribution of *tet *A and *tet *B genes among different cluster were different. It seems that *A. baumannii* molecular typing via PCR-based methods such as REP, could be beneficial in the differentiation of different strains of *A. baumannii* (30-33).

Despite the increased frequency of multidrug resistance among the *A. baumannii* strains from Iran, only a little information exists regarding the antimicrobial resistance of this Gram negative bacillus in Tehran, Iran. The results of this study confirms those of the previous study which was carried out only on burn patients and which reported *tet *A and *tet* B as the most frequent mechanisms of tetracycline resistance among Iranian *A. baumannii* isolates. The identification of *tet *A and *tet *B in this study also confirms the wide geographical distribu-tion of these resistance genes among tetracycline resistant *A. baumannii* strains. The results show that surveillance for multidrug resistant *A. baumannii* should be maintained and careful infection control and cautious use of antibiotics must be taken into consideration.

## Ethics

This study received approval from Ilam University of Medical Sciences Ethics Committee, Ilam, Iran.

## References

[1] Peleg AY, Seifert H, Paterson DL (2008). Acinetobacter baumannii: emergence of a successful pathogen. Clin Microbiol Rev.

[2] Hanlon GW (2005). The emergence of multidrug resistant Acinetobacter species: a major concern in the hospital setting. Lett Appl Microbiol.

[3] Asadollahi K, Alizadeh E, Akbari M, Taherikalani M, Niakan M, Maleki A (2011). The role of bla(OXA-like carbapenemase) and their insertion sequences (ISS) in the induction of resistance against carbapenem antibiotics among Acinetobacter baumannii isolates in Tehran hospitals. Roum Arch Microbiol Immunol.

[4] Asadollahi K, Taherikalani M, Maleki A, Alizadeh E, Valadbaigi H, Soroush S (2011). Diversity of aminoglycoside modifying enzyme genes among multidrug resistant Acinetobacter baumannii genotypes isolated from nosocomial infections in Tehran hospitals and their association with class 1 integrons. Acta Microbiol Immunol Hung.

[5] Feizabadi MM, Fathollahzadeh B, Taherikalani M, Rasoolinejad M, Sadeghifard N, Aligholi M (2008). Antimicrobial susceptibility patterns and distribution of blaOXA genes among Acinetobacter spp. Isolated from patients at Tehran hospitals. Jpn J Infect Dis.

[6] Taherikalani M, Fatolahzadeh B, Emaneini M, Soroush S, Feizabadi MM (2009). Distribution of different carbapenem resistant clones of Acinetobacter baumannii in Tehran hospitals. New Microbiol.

[7] Soroush S, Haghi-Ashtiani MT, Taheri-Kalani M, Emaneini M, Aligholi M, Sadeghifard N, Pakzad I (2010). Antimicrobial resistance of nosocomial strain of Acinetobacter baumannii in Children's Medical Center of Tehran: a 6-year prospective study. Acta Med Iran.

[8] Akbari M, Niakan M, Taherikalani M, Feizabadi MM, Azadi NA, Soroush S (2010). Rapid identification of Iranian Acinetobacter baumannii strains by single PCR assay using BLA oxa-51 -like carbapenemase and evaluation of the antimicrobial resistance profiles of the isolates. Acta Microbiol Immunol Hung.

[9] Taherikalani M, emadi G, Geliani KN, Fatollahzadeh B, Soroush S, Feizabadi MM (2008). Emergence of multi and pan-drug resistance Acinetobacter baumannii carrying blaOXA-type -carbapenemase genes among burn patients in Tehran, Iran. Saudi Med J.

[10] Kalantari N, Taherikalani M, Parvaneh N, Mamishi S (2007). Etiology and antimicrobial susceptibility of bacterial septic arthritis and osteomyelitis. Iran J Public Health.

[11] Haghi-Ashteiani M, Sadeghifard N, Abedini M, Soroush S, Taheri-Kalani M (2007). Etiology and antibacterial resistance of bacterial urinary tract infections in children's medical center, Tehran, Iran. Acta Medica Iranica.

[12] Asadollahi P, Akbari M, Soroush S, Taherikalani M, Asadollahi K, Sayemiri K (2012). Antimicrobial resistance patterns and their encoding genes among Acinetobacter baumannii strains isolated from burned patients. Burns.

[13] Akbari M (2010). Rapid identification of Iranian Acinetobacter baumannii strains by single PCR assay using blaOXA-51-like carbapenemase and evaluation of the antimicrobial resistance profiles of the isolates. Acta Microbiologica et Immunologica Hungarica.

[14] CLSI ( 2009). Methods for dilution antimicrobial susceptibility tests for bacteria that grow aerobically; approved standard.

[15] Pajand O, Rezaee MA, Nahaei MR, Mahdian R, Aghazadeh M, Soroush MH (2013). Study of the carbapenem resistance mechanisms in clinical isolates of Acinetobacter baumannii: Comparison of burn and non-burn strains. Burns.

[16] Srinivasan VB, Rajamohan G, Pancholi P, Stevenson K, Tadesse D, Patchanee P (2009). Genetic relatedness and molecular characterization of multidrug resistant Acinetobacter baumannii isolated in central Ohio, USA. Ann Clin Microbiol Antimicrob.

[17] Vila J, Marcos MA, Jimenez de Anta MT (1996). A comparative study of different PCR-based DNA fingerprinting techniques for typing of the Acinetobacter calcoaceticus-A. baumannii complex. J Med Microbiol.

[18] Safari M, Saidijam M, Bahador A, Jafari R, Alikhani MY (2013). High Prevalence of Multidrug Resistance and Metallo-beta-lactamase (MbetaL) producing Acinetobacter Baumannii Isolated from Patients in ICU Wards, Hamadan, Iran. J Res Health Sci.

[19] Bahador A, Taheri M, Pourakbari B, Hashemizadeh Z, Rostami H, Mansoori N (2013). Emergence of rifampicin, tigecycline, and colistin-resistant Acinetobacter baumannii in Iran; spreading of MDR strains of novel International Clone variants. Microb Drug Resist.

[20] Ruzin A, Keeney D, Bradford PA (2007). AdeABC multidrug efflux pump is associated with decreased susceptibility to tigecycline in Acinetobacter calcoaceticus-Acinetobacter baumannii complex. J Antimicrob Chemother.

[21] Seifert H, Stefanik D, Wisplinghoff H (2006). Comparative in vitro activities of tigecycline and 11 other antimicrobial agents against 215 epidemiologically defined multidrug-resistant Acinetobacter baumannii isolates. J Antimicrob Chemother.

[22] Mezzatesta ML, Trovato G, Gona F, Nicolosi VM, Nicolosi D, Carattoli A (2008). In vitro activity of tigecycline and comparators against carbapenem-susceptible and resistant Acinetobacter baumannii clinical isolates in Italy. Ann Clin Microbiol Antimicrob.

[23] Navon-Venezia S, Leavitt A, Carmeli Y (2007). High tigecycline resistance in multidrug-resistant Acinetobacter baumannii. J Antimicrob Chemother.

[24] Salazar De Vegas EZ, Nievesm B, Ruiz M, Ruíz J, Vila J, María A (2007). Molecular epidemiology and characterization of resistance mechanisms to various antimicrobial agents in Acinetobacter baumannii isolated in Merida, Venezuela. Med Sci Monit.

[25] Pei G, Mao Y, Sun Y (2012). In vitro activity of minocycline alone and in combination with cefoperazone-sulbactam against carbapenem-resistant Acinetobacter baumannii. Microb Drug Resist.

[26] Denys GA, Callister SM, Dowzicky MJ (2013). Antimicrobial susceptibility among gram-negative isolates collected in the USA between 2005 and 2011 as part of the Tigecycline Evaluation and Surveillance Trial (T.E.S.T.). Ann Clin Microbiol Antimicrob.

[27] Guardabassi L, Dijkshoorn L, Collard JM, Olsen JE, Dalsgaard A (2000). Distribution and in-vitro transfer of tetracycline resistance determinants in clinical and aquatic Acinetobacter strains. J Med Microbiol.

[28] Chopra I, Roberts M (2001). Tetracycline antibiotics: mode of action, applications, molecular biology, and epidemiology of bacterial resistance. Microbiol Mol Biol Rev.

[29] Ribera A, Ruiz J, Vila J (2003). Presence of the Tet M determinant in a clinical isolate of Acinetobacter baumannii. Antimicrob Agents Chemother.

[30] Mak JK, Kim MJ, Pham J, Tapsall J, White PA (2009). Antibiotic resistance determinants in nosocomial strains of multidrug-resistant Acinetobacter baumannii. J Antimicrob Chemother.

[31] Mammina C, Palma DM, Bonura C, Aleo A, Fasciana T, Sodano C (2012). Epidemiology and clonality of carbapenem-resistant Acinetobacter baumannii from an intensive care unit in Palermo, Italy. BMC Res Notes.

[32] Park S, Kim HS, Lee KM, Yoo JS, Yoo JI, Lee YS (2013). Molecular and epidemiological characterization of carbapenem-resistant Acinetobacter baumannii in non-tertiary Korean hospitals. Yonsei Med J.

[33] Andriamanantena TS, Ratsima E, Rakotonirina HC, Randrianirina F, Ramparany L, Carod J, etal (2010). Dissemination of multidrug resistant Acinetobacter baumannii in various hospitals of Antananarivo Madagascar. Ann Clin Microbiol Antimicrob.

